# Greatly enhanced light emission of MoS_2_ using photonic crystal heterojunction

**DOI:** 10.1038/s41598-017-16502-2

**Published:** 2017-11-27

**Authors:** Jiang-Tao Liu, Hong Tong, Zhen-Hua Wu, Jin-Bao Huang, Yun-Song Zhou

**Affiliations:** 1 College of Mechanical and electrical engineering, Guizhou Minzu University, Guiyang, 550025 China; 2 Department of Physics, Nanchang University, Nanchang, 330031 China; 3 Institute for Advancfed Study, Nanchang University, Nanchang, 330031 China; 4 Key Laboratory of Microelectronic Devices and Integrated Technology, Institute of Microelec-tronics, Chinese Academy of Sciences, Beijing, 100029 China; 5 Department of Physics, Capital Normal University, Beijing, 100037 China

## Abstract

We present theoretical study on developing a one-dimensional (1D) photonic crystal heterojunction (h-PhC) that consists of a monolayer molybdenum disulfide (MoS_2_) structure. By employing the transfer matrix method, we obtained the analytical solution of the light absorption and emission of two-dimensional materials in 1D h-PhC. Simultaneously enhancing the light absorption and emission of the medium in multiple frequency ranges is easy as h-PhC has more modes of photon localization than the common photonic crystal. Our numerical results demonstrate that the proposed 1D h-PhC can simultaneously enhance the light absorption and emission of MoS_2_ and enhance the photoluminescence spectrum of MoS_2_ by 2–3 orders of magnitude.

## Introduction

Two-dimensional (2D) transition metal dichalcogenides (TMDCs), such as MoS_2_ and WSe_2_, are direct-gap semiconductor 2D materials with excellent optical properties and are thus considered one of the best materials for future optoelectronic devices^1–7^. The light absorption and emission of 2D TMDCs per unit mass are much higher than that of traditional semiconductor materials. 2D TMDCs typically have a thickness of less than 1 nm, and their light absorption and emission are weak, thus limiting their applications in optoelectronic devices. However, due to the benefits of the ultra-thin channel, 2D TMDCs can be combined with optical microstructures, such as photonic crystals, microcavities, and surface plasmas, which effectively enhance the light absorption^8–31^ and emission^8,9,32–41^ due to the optical localization in these structures. Lien *et al*.^8^ and Serkan *et al*.^9^ used surface plasmas or optical multilayers to enhance the light absorption and emission of MoS_2_ or WSe_2_, thus enhanced the photoluminescence (PL) of MoS_2_ or WSe_2_ by 10–30 times.

To further enhance the light emission and absorption of 2D TMDCs, we investigated the effect of photonic crystal heterojunction (h-PhC) on the light absorption and emission of MoS_2_. Similar to the semiconductor heterojunction, h-PhC comprises photonic crystals (PhC) with different lattice constants or shapes^42^. Earlier studies have found that h-PhC that comprise different PhCs can obtain strong light localization in several frequency ranges^42,43^. On the basis of these findings, one can propose a h-PhC that is formed by different PhCs at intervals to form a multimode high-speed optical waveguide.

Thus, if 2D TMDCs are combined with h-PhC, the strong light localization of h-PhC in multiple frequency ranges can simultaneously enhance the light emission and absorption of 2D TMDCs. We therefore conducted a detailed study on a h-PhC which consists of 1D PhCs with two kinds of crystal lattices that form an h-PhC microcavity structure. To thoroughly understand the light absorption and emission in h-PhC, we first identified the analytical solution of the light absorption and emission of MoS_2_ in h-PhC. The findings indicate that h-PhC can enhance the light absorption and emission of MoS_2_ and enhance the PL spectrum of MoS_2_ by 2–3 orders of magnitude, which has a promising prospect and important application value in fluorescent probe, 2D LED, etc. The analytical solution can be used not only for the light absorption and emission in h-PhC but also for the calculation of other 1D PhC-2D materials composite structures.

## Model and Theory

**Figure 1:**
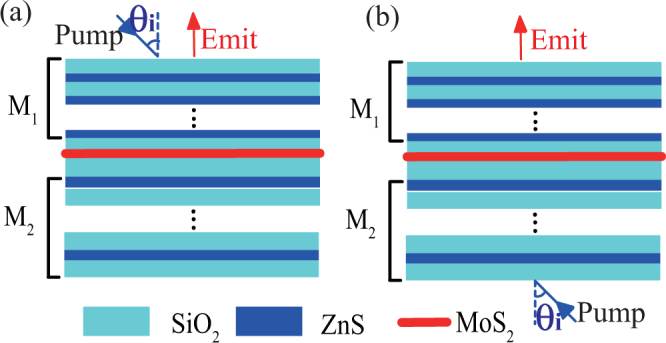
Schematic of h-PhC structure. (**a**) Pump light and outgoing light on the same side; (**b**) pump light and outgoing light on different sides.

The structure of h-PhC is shown in Fig. 1, i.e., ${({A}_{1}{B}_{1})}^{{N}_{1}}{C}_{1}M{C}_{2}{({B}_{2}{A}_{2})}^{{N}_{2}}$ structure. ${({A}_{1}{B}_{1})}^{{N}_{1}}$ and ${({B}_{2}{A}_{2})}^{{N}_{2}}$ layers constitute the two distributed Bragg reflectors (DBRs), where *N*_1_ and *N*_2_ are the numbers of cycles. The *A*_1_ and *A*_2_ layers are made of SiO_2_ with the permittivity ${n}_{Si{O}_{2}}=1.4923+0.81996\lambda {^{\prime} }^{2}/(\lambda {^{\prime} }^{2}-{0.10396}^{2})-0.01082\lambda {^{\prime} }^{2}$ ^44^. $\lambda ^{\prime} =\lambda \times {10}^{6}$, *λ* is wavelength of the input light beams, and the thicknesses of the *A*_1_ and *A*_2_ layers are *λ*_10_/(4 × 1.53) and *λ*_20_/(4 × 1.53), respectively. *λ*_10_ and *λ*_20_ are the center wavelengths of the the upside PhC and the bottom PhC, respectively. *B*_1_ and *B*_2_ layers are composed of ZnS with the permittivity ${n}_{ZnS}=8.393+$ $\mathrm{0.14383/(}\lambda {^{\prime} }^{2}-{0.2421}^{2})+\mathrm{4430.99/(}\lambda {^{\prime} }^{2}-{36.72}^{2})$ ^45^. The thicknesses of the *B*_1_ and *B*_2_ layers are *λ*_10_/(4 × 2.4) and *λ*_20_/(4 × 2.4). The *C*_1_ and *C*_2_ layers are made of SiO_2_, and their thicknesses are ${d}_{{C}_{1}}$ and ${d}_{{C}_{2}}$, respectively. The M layer is the MoS_2_ layer. Its thickness is 0.65 nm.

To model the absorption of MoS_2_ in this structure, the transfer matrix method is used first^46,22^. In the *l*-th layer, the electric field of the TE mode light beam with incident angle *θ*_*t*_ is given by

1 $${{\bf{E}}}_{l}(x,y)=[{A}_{l}{e}^{i{k}_{x}(x-{x}_{l})}+{B}_{l}{e}^{-i{k}_{x}(x-{x}_{l})}]{e}^{i{k}_{y}y}{{\bf{e}}}_{z},$$

where *k*_*l*_ = *k*_*lr*_ + *ik*_*li*_ is the wave vector of the incident light, **e**_*z*_ is the unit vectors in the z direction, and *x*_*i*_ is the position of the *l*-th layer in the x direction. And the magnetic field of the TM mode in the *l*-th layer is given by

2 $${{\bf{H}}}_{l}(x,y)=[{A}_{l}{e}^{i{k}_{x}(x-{x}_{l})}+{B}_{l}{e}^{i{k}_{x}(x-{x}_{l})}]{e}^{i{k}_{y}y}{{\bf{e}}}_{z},$$

The electric (magnetic) fields of TE (TM) mode in the (*l* + 1)-th and *l*-th layer are related by the matrix utilizing the boundary condition

3 $$(\begin{array}{c}{A}_{l+1}\\ {B}_{l+1}\end{array})=(\begin{array}{cc}\frac{{\gamma }_{l}+{\gamma }_{l+1}}{2{\gamma }_{l+1}}{e}^{i{k}_{x}{d}_{l}} & \frac{{\gamma }_{l+1}-{\gamma }_{l}}{2{\gamma }_{l+1}}{e}^{-i{k}_{x}{d}_{l}}\\ \frac{{\gamma }_{l+1}-{\gamma }_{l}}{2{\gamma }_{l+1}}{e}^{i{k}_{x}{d}_{l}} & \frac{{\gamma }_{l}+{\gamma }_{l+1}}{2{\gamma }_{l+1}}{e}^{-i{k}_{x}{d}_{l}}\end{array})(\begin{array}{c}{A}_{l}\\ {B}_{l}\end{array})={T}^{l+1\leftarrow l}(\begin{array}{c}{A}_{l}\\ {B}_{l}\end{array}),$$

where ${\gamma }_{l}=\frac{{k}_{lx}^{r}+i{k}_{lx}^{i}}{{\mu }_{l}(\omega )}$ $({\gamma }_{l}=\frac{{k}_{lx}^{r}+i{k}_{lx}^{i}}{{\varepsilon }_{l}(\omega )})$ for TE (TM) mode, *μ*_*l*_(*ω*) is the permeability, ${\varepsilon }_{l}(\omega )={\varepsilon }_{l}^{r}(\omega )+i{\varepsilon }_{l}^{i}(\omega )$ is the complex dielectric permittivity, and *d*_1_ is the thickness of the *l*-th layer. Thus, the fields in the (*l* + 1)-th layer are related to the incident fields by the transfer matrix

4 $$(\begin{array}{c}{A}_{l+1}\\ {B}_{l+1}\end{array})={T}^{l+1\leftarrow l}\cdot \cdot \cdot {T}^{2\leftarrow 1}{T}^{1\leftarrow 0}(\begin{array}{c}{A}_{0}\\ {B}_{0}\end{array})=(\begin{array}{cc}{T}_{11} & {T}_{12}\\ {T}_{21} & {T}_{22}\end{array})(\begin{array}{c}{A}_{0}\\ {B}_{0}\end{array}).$$

To thoroughly describe the light absorption and emission of MoS_2_ in h-PhC, improve the computational speed to optimize the structure, and help scholars who are not familiar with the transfer matrix method for computing, we obtained the analytical solutions of the light absorption and emission of MoS_2_ in h-PhC using the transfer matrix method. Since the transfer matrix of the electric fields of TE mode and the transfer matrix of magnetic fields of TM mode have the same form, we only show the analytical solution of the TE mode. First, for a N-period PhC in air, the transfer matrix can be written as^47^.

5 $$(\begin{array}{c}{A}_{0}\\ {B}_{0}\end{array})={M}_{N}(\begin{array}{c}{A}_{l+1}\\ {B}_{l+1}\end{array})=[\begin{array}{cc}\mathrm{1/}{t}_{N} & {r}_{N}^{\ast }/{t}_{N}^{\ast }\\ {r}_{N}/{t}_{N} & \mathrm{1/}{t}_{N}^{\ast }\end{array}](\begin{array}{c}{A}_{l+1}\\ {B}_{l+1}\end{array}),$$

where $\frac{1}{{t}_{N}}=\frac{1}{{t}_{0}}\frac{\sin \,N\beta }{\sin \,\beta }-\frac{\sin (N-1)\beta }{\sin \,\beta }$, $\frac{{r}_{N}}{{t}_{N}}=\frac{{r}_{0}}{{t}_{0}}\frac{\sin \,N\beta }{\sin \,\beta },\beta$ is the Bloch phase in each period, *t*_0_ and *r*_0_ are the transmission amplitude and reflection amplitude of the each period^47^. For the upper part PhC in the h-PhC, the right hand side is not air. By multiplying the transfer matrix of PhC to the C_1_ layer ${T}^{C\leftarrow P}({d}_{c}=\mathrm{0)}$ and the inverse transfer matrix of PhC to the air layer ${[{T}^{air\leftarrow P}({d}_{c}=\mathrm{0)}]}^{-1},$ we can get the transfer matrix of the upper part PhC

6 $$\begin{array}{rcl}({M}_{{N}_{1}}^{^{\prime} }) & = & {\{{M}_{{N}_{1}}{T}^{C\leftarrow P}({d}_{c}=\mathrm{0)}{[{T}^{air\leftarrow P}({d}_{c}=\mathrm{0)}]}^{-1}\}}^{-1}\\  & = & \frac{1}{2}(\begin{array}{cc}\zeta /{t}_{{N}_{1}}+\zeta ^{\prime} {r}_{{N}_{1}}^{\ast }/{t}_{{N}_{1}}^{\ast } & \zeta ^{\prime} {t}_{{N}_{1}}+\zeta {r}_{{N}_{1}}^{\ast }/{t}_{{N}_{1}}^{\ast }\\ \zeta {r}_{{N}_{1}}/{t}_{{N}_{1}}+\zeta ^{\prime} /{t}_{{N}_{1}}^{\ast } & \zeta ^{\prime} {r}_{{N}_{1}}/{t}_{{N}_{1}}+\zeta /{t}_{{N}_{1}}^{\ast }\end{array})\\  & = & [\begin{array}{cc}\mathrm{1/}{t}_{{N}_{1}}^{^{\prime} } & {r}_{{N}_{1}}^{^{\prime} \ast }/{t}_{{N}_{1}}^{^{\prime} \ast }\\ {r}_{{N}_{1}}^{^{\prime} }/{t}_{{N}_{1}}^{^{\prime} } & \mathrm{1/}{t}_{{N}_{1}}^{^{\prime} \ast }\end{array}],\end{array}$$

where $\zeta =1+\sqrt{{\varepsilon }_{c}}\,\cos \,{\theta }_{c},\zeta ^{\prime} \mathrm{=1}-\sqrt{{\varepsilon }_{c}}\,\cos \,{\theta }_{c},{\varepsilon }_{c}={\varepsilon }_{{c}_{1}}={\varepsilon }_{{c}_{2}}$ is the permittivity of C_1_ and C_2_ layers, ${\theta }_{c}={\theta }_{{c}_{1}}={\theta }_{{c}_{2}}$ is the propagation angle in the C_1_ and C_2_ layer. Similar, we can get the transfer matrix of the lower part PhC,

7 $$\begin{array}{rcl}{M}_{{N}_{2}}^{^{\prime} } & = & \{{[{T}^{C\leftarrow P}({d}_{c}=\mathrm{0)}]}^{-1}{T}^{air\leftarrow E}({d}_{c}=\mathrm{0)}\}{M}_{{N}_{2}}\\  & = & \frac{1}{2}(\begin{array}{cc}\zeta /{t}_{{N}_{2}}+\zeta ^{\prime} {r}_{{N}_{2}}/{t}_{{N}_{2}} & \zeta ^{\prime} /{t}_{{N}_{2}}^{\ast }+\zeta {r}_{{N}_{2}}^{\ast }/{t}_{{N}_{2}}^{\ast }\\ \zeta {r}_{{N}_{2}}/{t}_{{N}_{2}}+\zeta ^{\prime} /{t}_{{N}_{2}} & \zeta ^{\prime} {r}_{{N}_{2}}^{\ast }/{t}_{{N}_{2}}^{\ast }+\zeta /{t}_{{N}_{2}}^{\ast }\end{array})\\  & = & [\begin{array}{cc}\mathrm{1/}{t}_{{N}_{2}}^{^{\prime} } & {r}_{{N}_{2}}^{^{\prime} \ast }/{t}_{{N}_{2}}^{^{\prime} \ast }\\ {r}_{{N}_{2}}^{^{\prime} }/{t}_{{N}_{2}}^{^{\prime} } & \mathrm{1/}{t}_{{N}_{2}}^{^{\prime} \ast }\end{array}].\end{array}$$

The transfer matrix of the C_1_ layer is^11^.

8 $${M}_{f}({d}_{{C}_{1}})=[\begin{array}{cc}{e}^{-i{k}_{cx}{d}_{{C}_{1}}} & 0\\ 0 & {e}^{i{k}_{cx}{d}_{{C}_{1}}}\end{array}],$$

and the transfer matrix of the C_2_ layer is

9 $${M}_{f}({L}_{cav}-{d}_{{C}_{1}})=[\begin{array}{cc}{e}^{-i{k}_{cx}({L}_{cav}-{d}_{{C}_{1}})} & 0\\ 0 & {e}^{i{k}_{cx}({L}_{cav}-{d}_{{C}_{1}})}\end{array}],$$

where ${L}_{cav}={d}_{{C}_{1}}+{d}_{{C}_{2}}$ is the microcavity length, *k*_*cx*_ is the wave vector of the light in the C_1_ or C_2_ layer. Taking the approximation that ${e}^{i{k}_{Mx}{d}_{M}}\approx 1+i{k}_{Mx}{d}_{M}$, where *k*_*Mx*_ is the wave vector of the light in the MoS_2_ layer and *d*_*M*_ is the thickness of the and MoS_2_ layer, the transfer matrix of the MoS_2_ layer is

10 $${M}_{Mo{S}_{2}}={[{T}^{C\leftarrow Mo{S}_{2}}{T}^{Mo{S}_{2}\leftarrow C}(d=\mathrm{0)}]}^{-1}=[\begin{array}{cc}1-{\eta }_{1} & -{\eta }_{2}\\ {\eta }_{2} & 1+{\eta }_{1}\end{array}],$$

where ${\eta }_{1}=i{k}_{Mx}{d}_{M}[(\sqrt{{\varepsilon }_{Mo{S}_{2}}/{\varepsilon }_{c}}\cos \,{\theta }_{Mo{S}_{2}}/\cos \,{\theta }_{c})+(\sqrt{{\varepsilon }_{c}/{\varepsilon }_{Mo{S}_{2}}}\cos \,{\theta }_{c}/\cos \,{\theta }_{Mo{S}_{2}})]\mathrm{/2}$, and ${\eta }_{2}=i{k}_{Mx}{d}_{M}[(\sqrt{{\varepsilon }_{Mo{S}_{2}}/{\varepsilon }_{c}}$ $\cos \,{\theta }_{Mo{S}_{2}}/\cos \,{\theta }_{c})-(\sqrt{{\varepsilon }_{c}/{\varepsilon }_{Mo{S}_{2}}}\cos \,{\theta }_{c}/\cos \,{\theta }_{Mo{S}_{2}})]\mathrm{/2}$. Thus, the total transfer matrix of the *C*_1_, *C*_2_, and MoS_2_ layer is

11 $${M}_{f}({d}_{{C}_{1}}){M}_{Mo{S}_{2}}{M}_{f}(L-{d}_{{C}_{1}})=[\begin{array}{cc}(1-{\eta }_{1}){e}^{-ikL} & -{\eta }_{2}{e}^{ik(L-2{d}_{{C}_{1}})}\\ {\eta }_{2}{e}^{-ik(L-{d}_{{C}_{1}})} & (1+{\eta }_{1}){e}^{ikL}\end{array}].$$

The total transfer matrix of the h-PhC is

12 $$M={M}_{{N}_{1}}^{^{\prime} }{M}_{f}({d}_{{C}_{1}}){M}_{Mo{S}_{2}}{M}_{f}(L-{d}_{{C}_{1}}){M}_{{N}_{2}}^{^{\prime} }.$$

We can get the matrix element

13 $${M}_{11}=[(1-{\eta }_{1})/{\phi }_{1}{t}_{{N}_{1}}^{^{\prime} }-{\eta }_{2}{\phi }_{2}{r}_{{N}_{2}}^{^{\prime} }/{t}_{{N}_{1}}^{^{\prime} }+{\eta }_{2}{r}_{{N}_{1}}^{^{\prime} \ast }/{\phi }_{2}{t}_{{N}_{1}}^{^{\prime} \ast }+(1+{\eta }_{1}){\phi }_{1}{r}_{{N}_{2}}^{^{\prime} }{r}_{{N}_{1}}^{^{\prime} \ast }/{t}_{{N}_{1}}^{^{\prime} \ast }]/{t}_{{N}_{2}}^{^{\prime} },$$

and

14 $${M}_{21}=[(1-{\eta }_{1}){r}_{{N}_{1}}^{^{\prime} }/{\phi }_{1}{t}_{{N}_{1}}^{^{\prime} }-{\eta }_{2}{\phi }_{2}{r}_{{N}_{2}}^{^{\prime} }{r}_{{N}_{1}}^{^{\prime} }/{t}_{{N}_{1}}^{^{\prime} }+{\eta }_{2}/{\phi }_{2}{t}_{{N}_{1}}^{^{\prime} \ast }+(1+{\eta }_{1}){\phi }_{1}{r}_{{N}_{2}}^{^{\prime} }/{t}_{{N}_{1}}^{^{\prime} \ast }]/{t}_{{N}_{2}}^{^{\prime} },$$

where *φ*_1_ = *e* ^*ikL*^, ${\phi }_{2}={e}^{ik(L-2{d}_{{C}_{1}})}$. The transmittance of the h-PhC is $T={|\mathrm{1/}{M}_{11}|}^{2}$; the reflectance of the h-PhC is $R={|{M}_{21}/{M}_{11}|}^{2}$ ^11,47^ the absorption of MoS_2_ layer is ${A}_{Mo{S}_{2}}=1-R-T$.

The spontaneous emission of the monolayer MoS_2_ in the h-PhC can be treated as two emitted correlated wavepackets, upward (downward) propagating wave packet ${{\mathscr{P}}}_{u}$ (${{\mathscr{P}}}_{d}$). The emission wavepackets are partially transmitted and reflected by the two DBRs. The filed amplitude of the light emitted out from the exit DBR mirror is given by^48–50^.

15 $$\begin{array}{rcl}{E}_{DBRt}(t) & = & {t}_{t}{\mathscr{P}}(t)+{t}_{t}{r}_{b}{\mathscr{P}}(t-\frac{2{z}_{ol}}{c})\\  &  & +\,{t}_{t}({r}_{b}{r}_{t}){\mathscr{P}}(t-\frac{2{L}_{oc}}{c})\\  &  & +\,{t}_{t}({r}_{b}{r}_{b}{r}_{t}){\mathscr{P}}(t-\frac{2{z}_{ol}}{c}-\frac{2{L}_{oc}}{c})+\cdot \cdot \cdot ,\end{array}$$

where *r*_*t*_ and *t*_*t*_ are the reflection amplitude and transmission amplitude of the exit DBR mirror, respectively. *r*_*b*_ is the reflection amplitude of the back DBR mirror, *z*_*ol*_ is the distance between the monolayer MoS_2_ and back DBR mirror. When the pumping and outgoing lights are on the same side of the h-PhC, ${r}_{t}={r}_{{N}_{1}}^{^{\prime} },$ $\,{t}_{t}={t}_{{N}_{1}}^{^{\prime} },$ ${r}_{b}={r}_{{N}_{2}}^{^{\prime} }$, ${z}_{ol}={d}_{{C}_{2}}$; When the pumping and outgoing lights are on the opposite side of the h-PhC, ${r}_{t}={r}_{{N}_{2}}^{^{\prime} },$ ${t}_{t}={t}_{{N}_{2}}^{^{\prime} },$ ${r}_{b}={r}_{{N}_{1}}^{^{\prime} }$, ${z}_{ol}={d}_{{C}_{1}}$; ${\mathscr{P}}(t)$ is the electric amplitude against time for a single emission event (in either direction), ${L}_{oc}={n}_{c}{L}_{cav}={n}_{c}({d}_{{C}_{1}}+{d}_{{C}_{2}})$ is the optical length of microcavity, *n*_*c*_ is the permittivity of the C_1_ and C_2_ layer. By using the Fourier transform, the emitted radiation from the top DBR mirror in the frequency domain can be written as

16 $$\begin{array}{rcl}{E}_{DBRt}(\omega ) & = & \frac{{t}_{t}}{2\pi }{\int }_{-\infty }^{\infty }{\mathscr{P}}(t)\exp (i\omega t)dt\\  &  & +\,\frac{{t}_{t}{r}_{b}}{2\pi }{\int }_{-\infty }^{\infty }{\mathscr{P}}(t-\frac{2{z}_{ol}}{c})\exp (i\omega t)dt\\  &  & +\,\frac{{t}_{t}({r}_{b}{r}_{t})}{2\pi }{\int }_{-\infty }^{\infty }{\mathscr{P}}(t-\frac{2{L}_{oc}}{c})\exp (i\omega t)dt\\  &  & +\,\frac{{t}_{t}({r}_{b}{r}_{b}{r}_{t})}{2\pi }{\int }_{-\infty }^{\infty }{\mathscr{P}}(t-\frac{2{z}_{ol}}{c}-\frac{2{L}_{oc}}{c})\\  &  & \times \,\exp (i\omega t)dt+\cdot \cdot \cdot .\end{array}$$

Neglecting the changes of the spontaneous time, integral of Eq. (16), the emission intensity can be calculated by^48,49^.

17 $$\begin{array}{c}|{E}_{DBRt}(\lambda ){|}^{2}=\frac{1+{R}_{b}+2\sqrt{{R}_{b}}\,\cos (\frac{4\pi {n}_{c}{Z}_{OL}}{\lambda })}{1+{R}_{b}{R}_{t}-2\sqrt{{R}_{b}{R}_{t}}\,\cos (\frac{4\pi {n}_{c}{L}_{cav}}{\lambda })}\times {T}_{t}|{\mathscr{P}}(\lambda ){|}^{2},\end{array}$$

where ${T}_{t}=|{t}_{t}{|}^{2}$ and ${R}_{t}=|{r}_{t}{|}^{2}$ are the transmittance and reflectance of the exit DBR mirror, respectively, ${T}_{b}=|{t}_{b}{|}^{2}$ and ${R}_{b}=|{r}_{b}{|}^{2}$ are the the transmittance and reflectance of the back DBR mirror with the MoS_2_ TFT, respectively.

## Results

**Figure 2:**
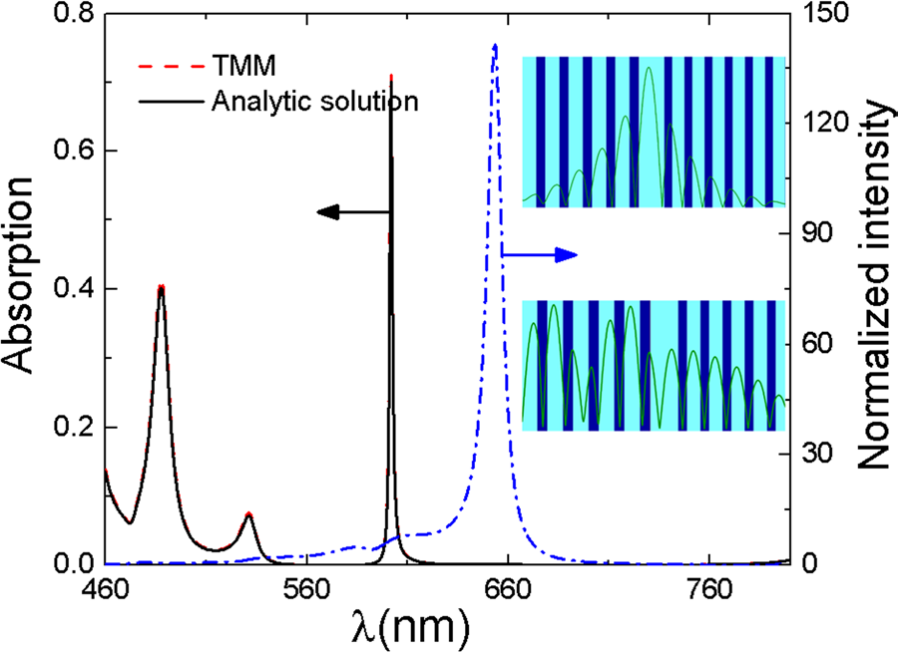
The absorption and relative radiation intensity of MoS_2_ when the pump and outgoing lights are on the same side of the h-PhC. The black solid line and the red dashed line are the calculation results of the analytical solution and the transfer matrix method, respectively. The upper and lower illustrations are the light field distribution at wavelengths of 488 and 602 nm, respectively.

We first calculated the absorption and the relative radiation intensity of MoS_2_ when the pumping and outgoing lights are on the same side of the h-PhC. The optimized parameters are as follows: *λ*_10_ = 730 nm, *λ*_20_ = 630 nm, ${d}_{{C}_{1}}=0\,{\rm{nm}}$, ${d}_{{C}_{2}}=214\,{\rm{nm}}$, *N*_1_ = 6, and *N*_2_ = 7. The incident angle is *θ*_*i*_ = 48°. The pumping light is in TE mode. The outgoing light is vertically emitted. The calculation results are shown in Fig. 2. Two strong absorption peaks emerge at wavelengths of 488 nm (consistent with the wavelength of the pump light used in the experiment^9^) and 602 nm. Quite small difference can be found between the numerical transfer matrix method and the calculation results from the analytical solution with the approximation ${e}^{i{k}_{Mx}{d}_{M}}\approx 1+i{k}_{Mx}{d}_{M}$. The optical wavelength of 602 nm is in the bandgap of the two PhCs with strong localization properties (upper illustration of Fig. 1) and strong absorption. The absorption can reach 0.7 or more, which is approximately 6 times larger than that without h-PhC. If the wavelength of the pumping light is 488 nm, it is only in the bandgap of the bottom PhC. The localization is weak. The absorption is 0.4, which is about 3 times larger than that without h-PhC. The emission efficiency is enhanced by 140 times due to the high reflectivity of the PhCs on both sides. Thus, when the lights are of 488 nm and 602 nm wavelengths, the PL is enhanced by approximately 420 and 840 times, respectively.

**Figure 3:**
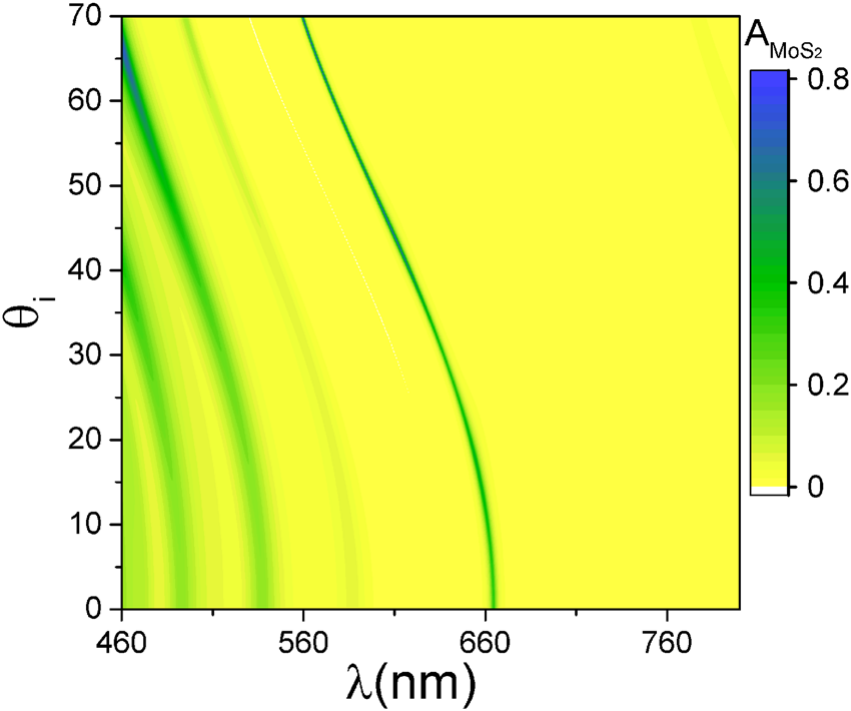
h-PhC MoS_2_ absorption changes as the incident angle and wavelength change.

The MoS_2_ absorption in h-PhC is sensitive to the incidence angle. The calculation results are shown in Fig. 3. The resonant wavelength of the microcavity satisfies the relation ship ${m}_{0}{\lambda }_{c}\mathrm{/2}={L}_{c}\,{\cos }\theta ^{\prime}$. *L*_c_ = *n*_*c*_ *d*_*c*_ is the microcavity optical path, *m*_0_ is a positive integer, and $\theta ^{\prime} =arcsin{\theta }_{i}/{n}_{c}$ is the propagation angle of light in the defective layer. Thus, when the incident angle increases, the resonance wavelength moves in the short-wave direction, the reflectivities of the PhCs on both sides increases, the travel path of light in MoS_2_ increases, and the maximum absorption can reach 0.8 or more. PL is enhanced by approximately 3 orders of magnitude.

We also calculated the positive incidence of the pumping light and the absorption and relative radiation intensity of MoS_2_ when the pumping and outgoing lights are on opposite sides of the h-PhC. According to the experiment^9^, in our calculation, the wavelength of the pumping light is 488 nm, and the wavelength of the outgoing light approaches 660 nm. We calculated the corresponding parameters by optimizing the h-PhC structure under different pumping light incidences as follows: when the pumping light is normally incident and the pumping and outgoing lights are on the same side of the h-PhC, *λ*_10_ = 580 nm, *λ*_20_ = 610 nm, ${d}_{{C}_{1}}=2\,{\rm{nm}}$, ${d}_{{C}_{2}}=214\,{\rm{nm}}$, *N*_1_ = 7, and *N*_2_ = 7. When the pumping light is normally incident and the pumping and outgoing lights are on opposite sides of the h-PhC, *λ*_10_ = 630 nm, *λ*_20_ 570 nm, ${d}_{{C}_{1}}=11\,{\rm{nm}}$, ${d}_{{C}_{2}}=203\,{\rm{nm}}$, *N*_1_ = 7, and *N*_2_ = 7. When the pumping light is obliquely incident and the pumping and outgoing lights are on opposite sides of the h-PhC, *λ*_10_ = 660 nm, *λ*_20_ = 580 nm, ${d}_{{C}_{1}}=0\,{\rm{nm}}$, ${d}_{{C}_{2}}=216\,{\rm{nm}}$, *N*_1_ = 7, *N*_2_ = 7, and *θ*_*i*_ = 30°.

**Figure 4:**
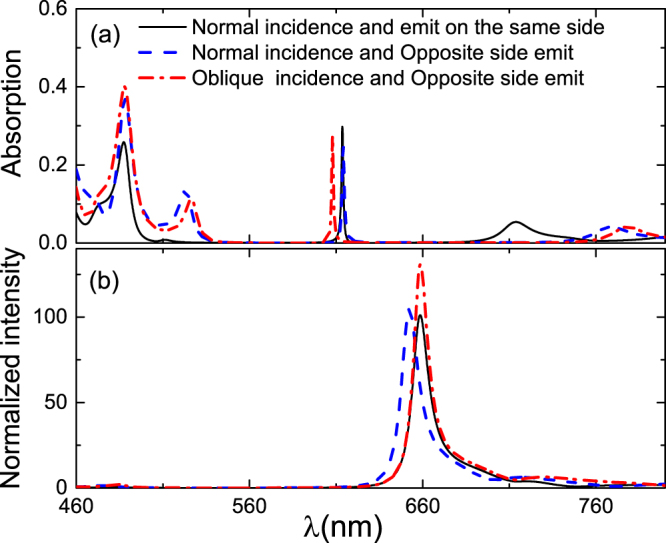
(**a**) Light absorption and (**b**) light emission of MoS_2_ in h-PhC under different pump lights. The black solid line: when the pump light is normally incident, the pump and outgoing lights are in the same side of the h-PhC; the blue dashed line: when the pump light is normally incident, the pump and outgoing lights are on opposite sides of the h-PhC; the red dash-dotted line: when the pump light is obliquely incident, the pump and outgoing lights are on opposite sides of the h-PhC.

The detailed calculation results are shown in Fig. 4. Regardless of whether the pumping and outgoing lights are on the same or different sides of the h-PhC, the absorption of MoS_2_ is higher when the pumping light is obliquely incident. A strong local touch in the vicinity of two wavelengths can be easily obtained as change in incidence angle can adjust the resonant wavelength. When the pumping light is normally incident and the pumping and outgoing lights are on opposite sides of the h-PhC, the absorption of MoS_2_ is high because if MoS_2_ in the microcavity structure obtains strong absorption and emission, the reflectivity of the rear reflector should be higher but the reflectivity of the front reflector should not be excessively high^19^. The bandgap width of the PhC is not big enough due to the large difference between the wavelengths of the pumping and outgoing lights. The pumping and outgoing lights on different sides realize this goal easily.

**Figure 5:**
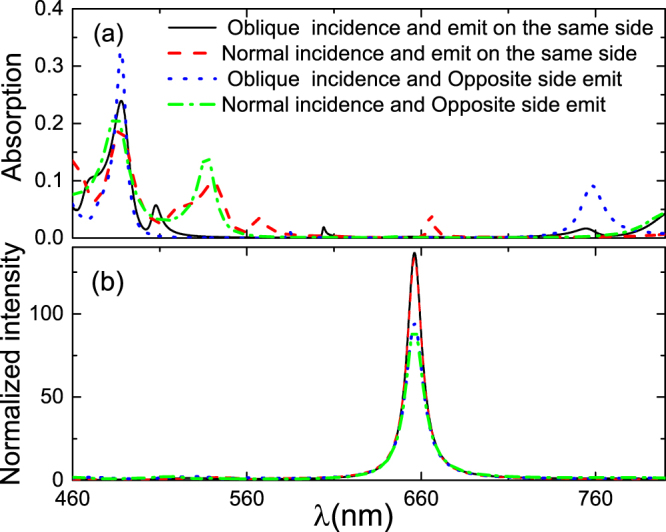
(**a**) Light absorption and (**b**) light emission of MoS_2_ in homojunction PhC under different pumping lights. The black solid line: when the pumping light is obliquely incident and the pumping and outgoing lights are on the same side; the red dashed line: when the pumping light is normally incident and the pumping and outgoing lights are on the same side; the blue dotted line: when the pumping light is obliquely incident and the pumping and outgoing lights are on opposite sides; the green dash-dotted line: when the pumping light is normally incident and the pumping and outgoing lights are on opposite sides.

For comparison, we calculated the light absorption and emission of MoS_2_ in a homojunction. The optimized structural parameters are as follows: when the pumping light is obliquely incident and the pumping and outgoing lights are on the same side, ${\lambda }_{10}={\lambda }_{20}=660\,{\rm{nm}}$, ${d}_{{C}_{1}}=0\,{\rm{nm}}$, ${d}_{{C}_{2}}=215\,{\rm{nm}}$, *N*_1_ = 5, *N*_2_ = 7, and *θ*_*i*_ = 42°. When the pumping light is normally incident and the pumping and outgoing lights are on the same side, ${\lambda }_{10}={\lambda }_{20}=680\,{\rm{nm}}$, ${d}_{{C}_{1}}=4\,{\rm{nm}}$, ${d}_{{C}_{2}}=210\,{\rm{nm}}$, *N*_1_ = 5, and *N*_2_ = 7. When the pumping light is obliquely incident and the pumping and outgoing lights are on opposite sides, ${\lambda }_{10}={\lambda }_{20}=670\,{\rm{nm}}$, ${d}_{{C}_{1}}=0\,{\rm{nm}}$, ${d}_{{C}_{2}}=214\,{\rm{nm}}$, *N*_1_ = 6, *N*_2_ = 5, and *θ*_*i*_ = 54°. When the pumping light is normally incident and the pumping and outgoing lights are on opposite sides: ${\lambda }_{10}={\lambda }_{20}=650\,{\rm{nm}}$, ${d}_{{C}_{1}}=0\,{\rm{nm}}$, ${d}_{{C}_{2}}=214\,{\rm{nm}}$, *N*_1_ = 6, and *N*_2_ = 5. The calculation results are shown in Fig. 5. These structures show that the localization of homojunction PhC is not excessive and increasing the light absorption and emission of MoS_2_ at the same time is difficult. When the light emission is strong, the light absorption is usually low, with an absorption of only approximately 0.2. When the pumping light is obliquely incident and the pumping and outgoing lights are on opposite sides, the absorption is the largest (approximately 0.33) but the outgoing light enhancement is low. If light emission is enhanced using longer PhC cycles than those used in the current study, the light absorption of MoS_2_ will decrease. However, this case does not happen in h-PhC.

Finally, we discuss the effect of light localization and the feasibility of the experiment.

The effect of light localization: We used the Q value to judge the strength of light localization. The larger the Q value, the higher the light localization and light absorption and emission intensity of MoS_2_. However, the higher the Q value, the smaller the full width at half maximum of the spectral line and the narrower the absorption and PL spectra. Excessively narrow absorption and emission spectra are not conducive to practical application. Moreover, when the Q value is high, the microcavity affects the transition time of the exciton. Thus, in Optimizing the parameters, we choose *N*_1_ ≤ 7 and *N*_2_ ≤ 7.

The feasibility of the experiment: PhC and 2D materials composite structures (particularly 2D materials-PhC microcavity) were created ^14–16^. Compared with the existing structure, this structure only changes the lattice constant of the upper and lower reflectors of the PhC microcavity. Therefore, the experiment is completely achievable.

## Conclusion

We studied the effect of 1D h-PhC on the light absorption and emission of monolayer MoS_2_ and obtained the solutions of both light absorption and emission in 1D PhC-2D materials composite structures. h-PhC has more models of photon localization than common PhC, which enhances the light emission and absorption of MoS_2_ simultaneously, and increases the PL spectrum of MoS_2_ by 2–3 orders of magnitude. When the pumping light is obliquely incident and the pumping and outgoing lights are on opposite sides of the h-PhC, it is easier to enhance the light absorption and emission of MoS_2_ at the same time. The analytical solution can be used not only for light absorption and emission in h-PhC but also applicable for other 1D PhC-2D materials composite structures. This study has a promising prospect and important application value in 2D material-based optoelectronic devices.

## Methods

The modified transfer matrix method is used to model the absorption of monolayer MoS_2_ in the photonic crystal micro-cavity.
